# Are Amyloid Fibrils RNA-Traps? A Molecular Dynamics Perspective

**DOI:** 10.3389/fmolb.2018.00053

**Published:** 2018-06-11

**Authors:** Massimiliano Meli, Maria Gasset, Giorgio Colombo

**Affiliations:** ^1^Consiglio Nazionale delle Ricerche, Instituto di Chimica del Riconoscimento Molecolare, Milan, Italy; ^2^Consejo Superior de Investigaciones Científicas, Instituto Química-Física “Rocasolano”, Madrid, Spain; ^3^Dipartimento di Chimica, Università degli Studi di Pavia, Pavia, Italy

**Keywords:** amyloids, molecular dynamics, RNA, molecular recognition, anti-amyloid drugs

## Abstract

The self-assembly of proteins and peptides into amyloids is a key feature of an increasing number of diseases. Amyloid fibrils display a unique surface reactivity endowing the sequestration of molecules such as MicroRNAs, which can be the active moiety of the toxic action. To test this hypothesis we studied the recognition between a model RNA and two different steric zipper spines using molecular dynamics simulations. We found that the interaction occurs and displays peptide-sequence dependence. Interestingly, interactions with polar zipper surfaces such as the formed by SNQNNF are more stable and favor the formation of β-barrel like complexes resembling the structures of toxic oligomers. These sequence-structure-recognition relationships of the two different assemblies may be exploited for the design of compounds targeting the fibers or competing with RNA-amyloid attachment

## Introduction

The self-assembly of peptides and proteins into amyloid aggregates is the common thread of a large number of degenerative diseases including Creutzfeldt-Jakob, Parkinson's, Alzheimer's, Amyotrophic Lateral Sclerosis, type 2 diabetes, among others (Chiti and Dobson, 2006, 2017). The nature, structure, and functional mechanism of these assemblies are still partially elusive (Riek and Eisenberg, 2016). Recent findings have shown that amyloid fibrils display a unique surface reactivity endowing the aberrant sequestration of distinct molecules and secondary nucleation events (Cascella et al., 2016; Boulay et al., 2017; Gaspar et al., 2017; Monahan et al., 2017). Interestingly, RNA has been hypothesized as a cofactor for the pathogenicity of both toxic amyloids and prions (Kellings et al., 1993; Safar et al., 2005; Vieira et al., 2011; Kucherenko and Shcherbata, 2018). In this context, it is tempting to speculate on the role of amyloids as RNA traps, which may hijack or misplace RNA-encoded messages. RNA and amyloid aggregates have indeed been found also in the material transported by exosomes (Coleman and Hill, 2015; Ngolab et al., 2017; Yuyama and Igarashi, 2017). Therefore, understanding the molecular details of the process of RNA-amyloid recognition may be important from an experimental as well as a theoretical perspective.

While significant efforts and important results have been obtained for the characterization of amyloid structures using diverse techniques, to the best of our knowledge no molecular resolution model exists for amyloid-nucleic acid complexes. To start investigating this at an atomic level, we have little choice but to turn to theoretical and simulative approaches, which have been proved effective in elucidating the mechanistic details of folding and unfolding as well as of many pathogenic processes (De Mori et al., 2004; Pontiggia et al., 2007; Meli et al., 2008; Moroni et al., 2015). This approach can be enabled by the knowledge of the atomic structures of different self-assembling segments of amyloid fibrils, termed steric zippers, which reveal a common motif for the spine whereby a pair of fibrillar beta-sheets is held together by side-chain interdigitation (Sawaya et al., 2007; Apostol et al., 2010; Gallagher-Jones et al., 2018).

To investigate whether RNA interacts with amyloid fibril surfaces and to garner insight into the chemical rules at the basis of recognition we have used a simple RNA model sequence and the surfaces of two aggregates. As RNA we choose the UCCU sequence given its presence in small RNAs such as miR23a (GGGGU***UCCU***GGGGAUGGGAUUU), miR378a (C***UCCU***GAC***UCCA***GG***UCCU***GUGU), and miR344 (AGUCAGGC***UCCU***GGCUAGAU***UCCA***GG) released in exosomes of prion-infected cells (Bellingham et al., 2012). In this case, the tetramer is to be considered as a minimal model to probe the main determinants of amyloid-RNA interaction. As models of surfaces we choose those generated by the steric zippers assembly of regions 127-132 (GYMLGS) and 170-175 (SNQNNF) of human PrP, which are relevant to disease phenotype and species barrier, respectively (Sawaya et al., 2007; Apostol et al., 2010). Interestingly, the latter sequence represents a so-called polar zipper, while the former does not. By means of multiple runs of MD simulations, we found specific patterns of interactions of the RNA model sequence on the surfaces, which may in turn be used for the development of novel pharmacophore models for drug database screening.

## Materials and methods

### MD simulations

The conformational analysis of the RNA tetramer free in solution was started from the completely extended conformation of the nucleic acid. The crystal models for SNQNNF and GYMLGS sequences were built by using the cell unit information as deposited in pdb database with codes 2OL9 and 3NHC respectively (Sawaya et al., 2007; Apostol et al., 2010). The unit cell was replicated along the X, Y, and Z directions so that the resulting dimension of the surface available for binding was much larger than the length if a completely extended RNA model fragment. The unit cell replication was carried out using the program Chimera (Pettersen et al., 2004). All the geometric information about the system used during the successive phase of this work is summarized in Table 1. The peptide crystals were simulated alone for 200 ns and observed to be stable in these conditions in the absence of RNA.

**Table 1:** Summary of the sequence and structural properties of the amyloid peptide crystals.

**Sequence^a^**	**Direction^b^**	**Unit cell dimension^c^ [A, degree]**	**Times^d^**	**Crystal dimension^e^ [A]**	**Final simulation box dimension [A]**	**Total number of atoms**
GYMLGS (Sc 0.74)	X	*a* 9.439, *b* 17.792, *c* 44.561 a 90.00, b 90.00, c 90.00	2, 4, 4	18.8, 71.1, 178.2	92.9, 71.1, 178.2	118431
Space group: P2_1_2_1_2_1_	Y		8, 2, 3	75.5, 35.5, 133.6	75.5, 135.4, 133.6	140372
	Z		8, 7, 1	75.5, 124.5, 44.5	75.5, 124.5, 177.7	173952
SNQNNF (Sc 0.90)	X	*a* 14.002, *b* 4.879, *c* 15.100 a 75.23, b 75.88, c 78.89	2, 14, 10	28.0, 68.3, 151.0	133.0, 68.3, 151.0	125781
Space group: P1	Y		6, 3, 6	84.0, 14.6, 90.6	84.0, 73.1, 90.6	53813
	Z		10, 10, 2	140.0, 48.7, 30.2	140.0, 48.7, 105.7	73748

a *Sc, shape complementarity (Sawaya et al., 2007; Apostol et al., 2010)*.

b *The direction respect the crystal axis along the RNA was placed*.

c *Unit cell shape parameters*.

d *Number of times that the unit cell was replicated along the direction X Y and Z*.

e *The final crystal dimension obtained through the asymmetric unit replication process*.

All MD simulations and the successive energetic and conformational analyses were carried out using the Amber 16—AmberTools 16 suite of programs (Case et al., 2017). We used the Amber ff14SB for the peptides, and the Amber ff99bsc0_chiOL3 force field for the small RNA tetramer (Pérez et al., 2007; Zgarbova et al., 2011). Water solvation was modeled via the TIP3P water model. Counter ions was added by using “AddToBox” program present in AmberTools, to reach an ionic strength around 100 mM.

Each system listed in Table 1 was simulated with three independent runs of 200 ns each (for a total of 600 ns). The third simulation was started from the last frame of the first one. The simulation protocol used for all the system started with an energy minimization with Amber in explicit solvent with a steepest descent method (3 × 10^3^ steps), followed by a run with conjugate gradient algorithm (7 × 10^3^ steps). After minimization, the production simulations were conducted with isotropic position scaling (ntp = 1) and the temperature was kept constant using a weak-coupling algorithm (Berendsen et al., 1984). For each simulation were collected 50,000 frames every 2 ps.

After completing the simulations of the complexes, we combined all the trajectories of the RNA molecules and used the Gromos clustering algorithm (Daura et al., 1999) to extract the principal representative conformations of the RNA model when bound to the amyloid fibrils. Briefly, from the combined trajectories of RNA, the RMSD of atom positions between all pairs of structures, calculated on the backbone atoms, was determined. As described by Daura et al. in the original paper (Daura et al., 1999), for each structure the number of other structures for which the RMSD was below 0.2 nm was calculated. The structure with the highest number of neighbors was taken as the center of a cluster, and combined with all its structural neighbors within the cutoff a defined the (first) cluster. The structures of this cluster were eliminated from the pool of structures and the process iterated until the inclusion of all structures in clusters.

### MMPBSA energy calculations

The interaction energies between the crystal and RNA copies were calculated using the Molecular Mechanics/Poisson Boltzmann Surface Area (MM/PBSA) method as implemented in Amber 16 (Case et al., 2017). We extracted the last 2,500 frame from the third simulation of each system. Interactions with RNA were considered for all peptides within a 12 Å range from the RNA fragment. We notice here that all analyses and calculations are run on the various conformations of RNAs in contact with the peptides. Given the obvious time limitations of our simulations, we observed no unbinding events or reversible binding-unbinding phenomena.

## Results and discussion

### RNA-amyloid model interactions

**Figure 1:**
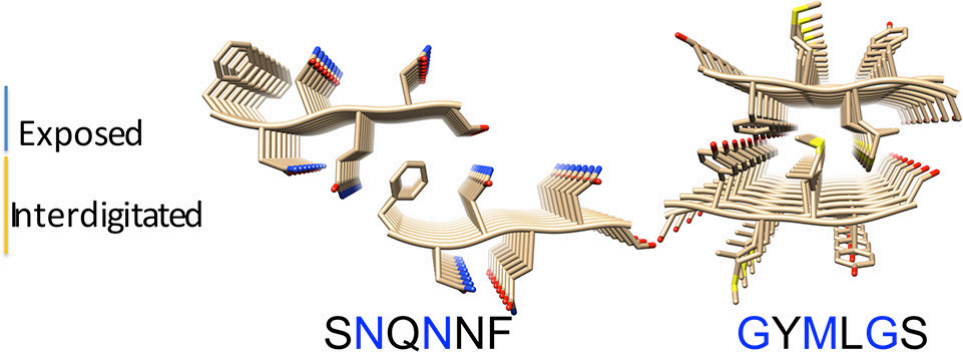
Amyloid fibril models. The structural organization of the SNQNNF and GYMLGS sequences from which the fibril models have been built were taken from Sawaya et al. (2007) and Apostol et al. (2010). Residues with solvent exposed side chains are depicted in blue.

**Figure 2:**
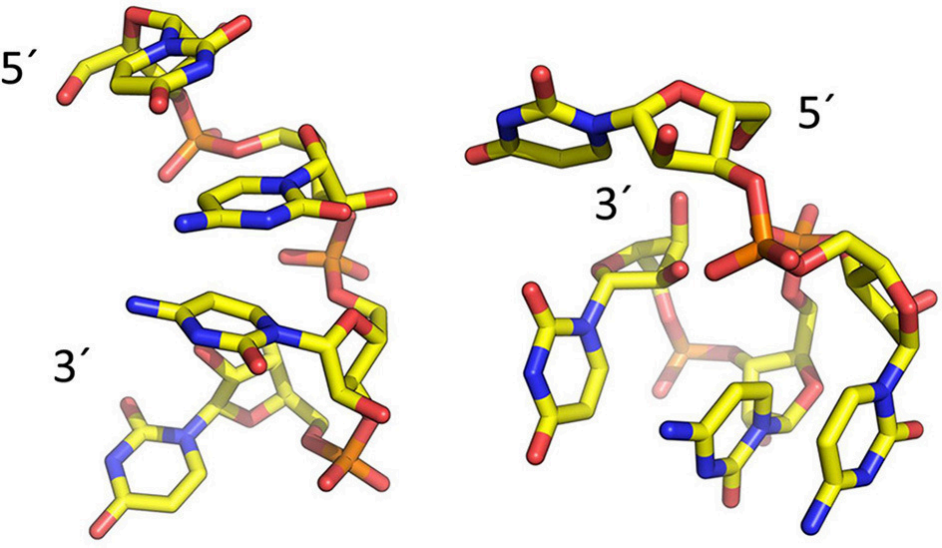
Representative structures of the two main conformational clusters of UCCU RNA model obtained by MD simulations. The 5′ and 3′ ends are indicated.

To model the amyloid aggregates we used the basic unit of the steric zippers determined by the Eisenberg's group (PDB codes 2OL9 and 3NHC). Each basic unit cell was replicated along two directions, while explicit water was added along the third direction providing simulation boxes with the dimensions reported in Table 1. Figure 1 shows the constructed model structures and the differences in the repetitive pattern of solvent-exposed side chains. To gain insights the conformational preferences of the nucleic acid stretch, preliminary MD simulations of the UCCU RNA tetramer (2 replicas of 200 ns simulations) isolated in solution were first carried out. Cluster analysis of the combined trajectories identified two major structural ensembles. Interestingly, when free in solution intercalated RNA structures, known to be either absent or present at very low percentage, represent only about 5% of all observed conformations (Tubbs et al., 2013; Bergonzo et al., 2015; Condon et al., 2015). It is however reasonable to hypothesize that this situation may change once the nucleic-acid tetramer contacts the amyloid crystal. The representative structures of the two main clusters were used as initial structures to investigate interactions with amyloids (Figure 2).

**Figure 3:**
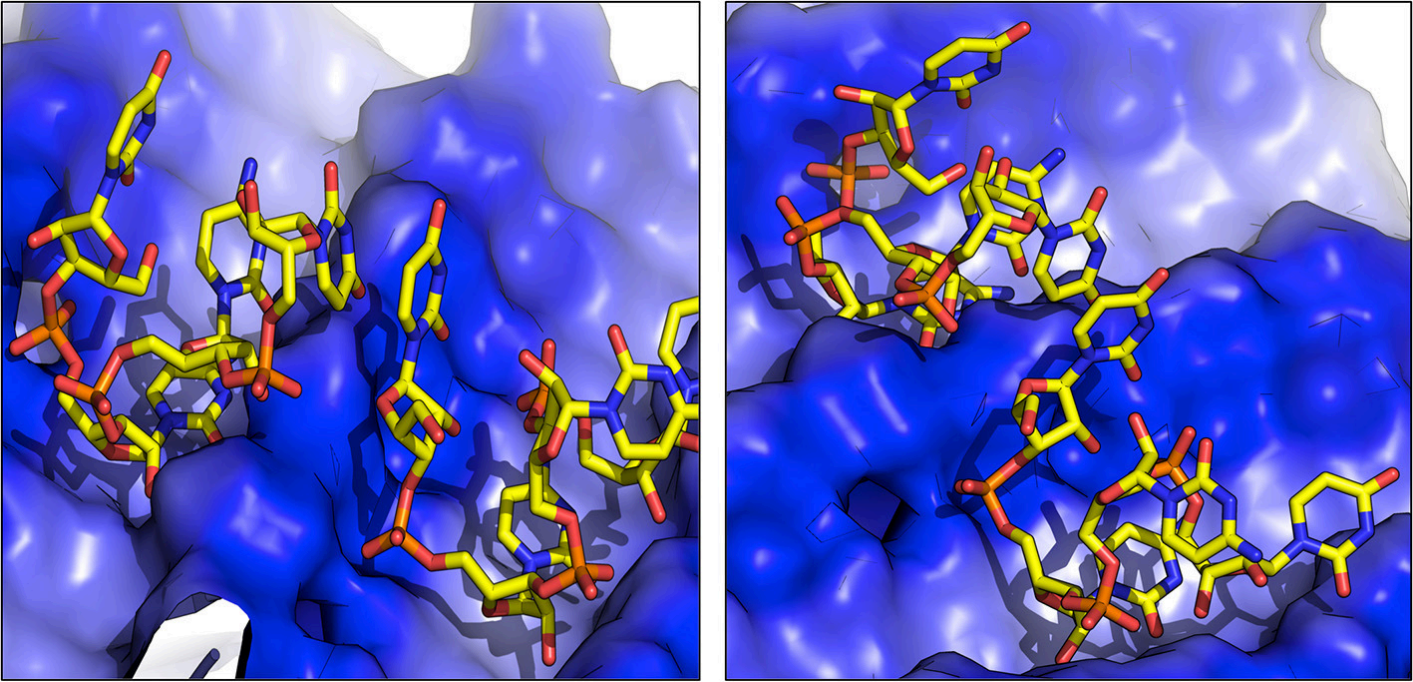
Representative structures of RNA dimers formed on the surface of fibrils. The blue color indicates the positively charged surface in contact with RNA.

For each of the interaction simulations, four different RNA chains (two representing the first structural cluster and two representing the second cluster) were randomly placed in the bulk water layer. Interestingly, once docked onto the amyloid the RNA molecules may shift their position on the surface, and undergo conformational changes. In some cases, the formation of dimers was observed (Figure 3). Considering the mobility of the nucleotide on the amyloid surface, 2,500 representative snapshots for aggregate-RNA interactions were extracted from the last 200 ns of each simulation.

**Figure 4:**
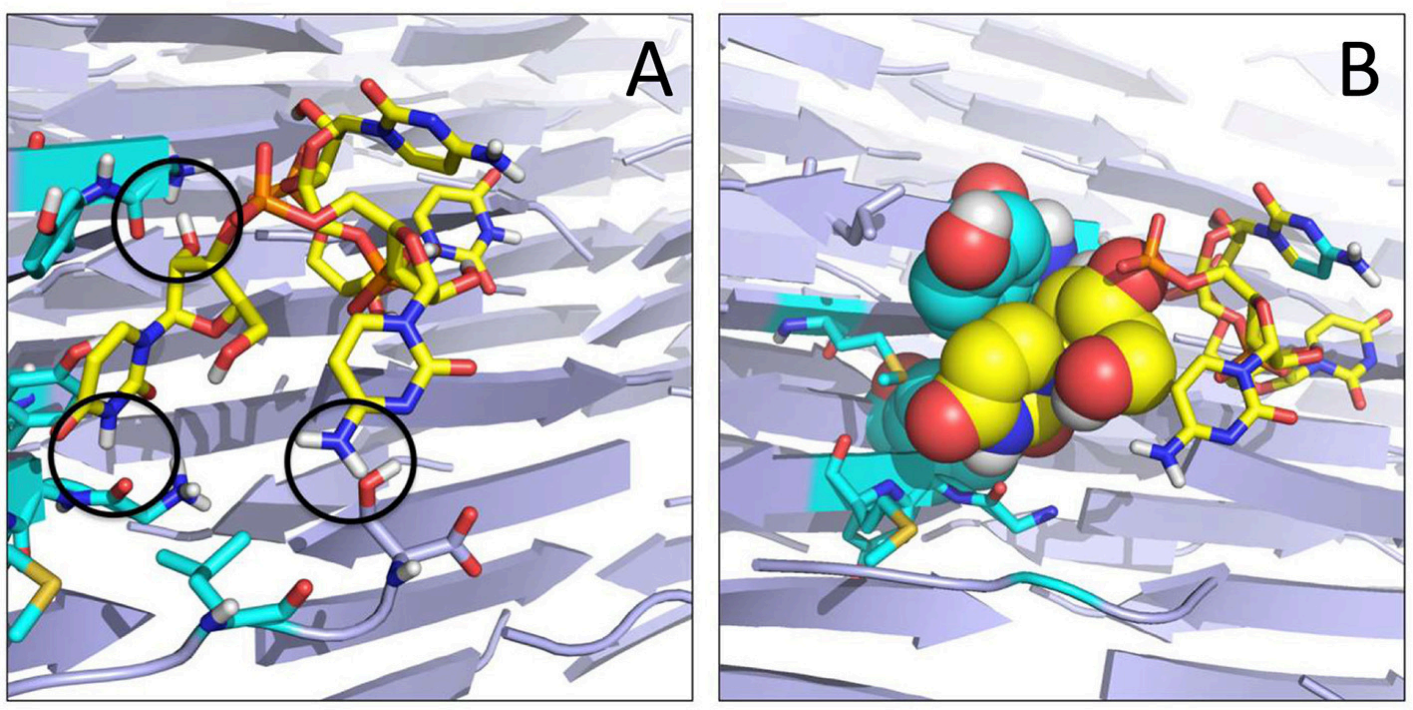
Organization of the RNA tetramer on the surface of the GYMLGS aggregate. **(A)** RNA forms different h-bonds on the surface (black circles). **(B)** Detail of the packing of U5 (yellow) with Y residues from the peptide assembly.

Each of the representative structures was next used to calculate the energetics of the interaction by means of the MMPBSA approach (see Materials and Methods). Within the limitations of our model (unbinding events and reversible binding-unbinding were not observed, and MMPBSA relies on approximations such as the neglect of conformational entropies) (Genheden and Ryde, 2015), the data reported in Table 2 shows that the two amyloid sequences have different affinities for the RNA model stretch, particularly if the latter approaches from the YZ or XY surfaces. The polar SNQNNF-zipper shows much larger affinities (lower free energies) than the GYMLGS-spine. This behavior is qualitatively paralleled by the increased mobility of the RNA stretch on the GYMLGS aggregate. Interestingly, as the RNA molecules target the aggregate from the XZ surface, similar trends between the two sequences are observed. From the qualitative point of view, approaching from the XZ surface favors H-bonding interactions between the backbones/side chains of peptides and RNA, whereas the approach of RNA ligands from the YZ or XY directions favors electrostatic and side-chain packing interactions with the nucleotides (See Figure 4).

**Table 2:** 3-dimensional energetics of the amyloid-RNA structures.

**Sequence**	**Interaction surface**	**Fragment 1 (kcal/mol)**	**Fragment 2 (kcal/mol)**	**Fragment 3 (kcal/mol)**	**Fragment 4 (kcal/mol)**
GYMLGS	YZ	−19.2 ± 8.3	−19.9 ± 7.5	−36.4 ± 10.5	−20.3 ± 13.4
	XZ	−31.0 ± 7.8	−14.9 ± 11.0	−12.5 ± 6.8	−13.8 ± 8.5
	XY	−1.1 ± 7.6	−26.7 ± 16.3	−14.5 ± 16.7	4.7 ± 4.2
SNQNNF	YZ	−39.9 ± 11.4	−48.8 ± 12.6	−36.4 ± 20.0	−56.1 ± 21.3
	XZ	−16.7 ± 10.6	−14.7 ± 9.5	−23.5 ± 17.5	−24.3 ± 18.0
	XY	−35.1 ± 11.0	−52.0 ± 16.7	−61.1 ± 25.0	−73.1 ± 22.0

**Figure 5:**
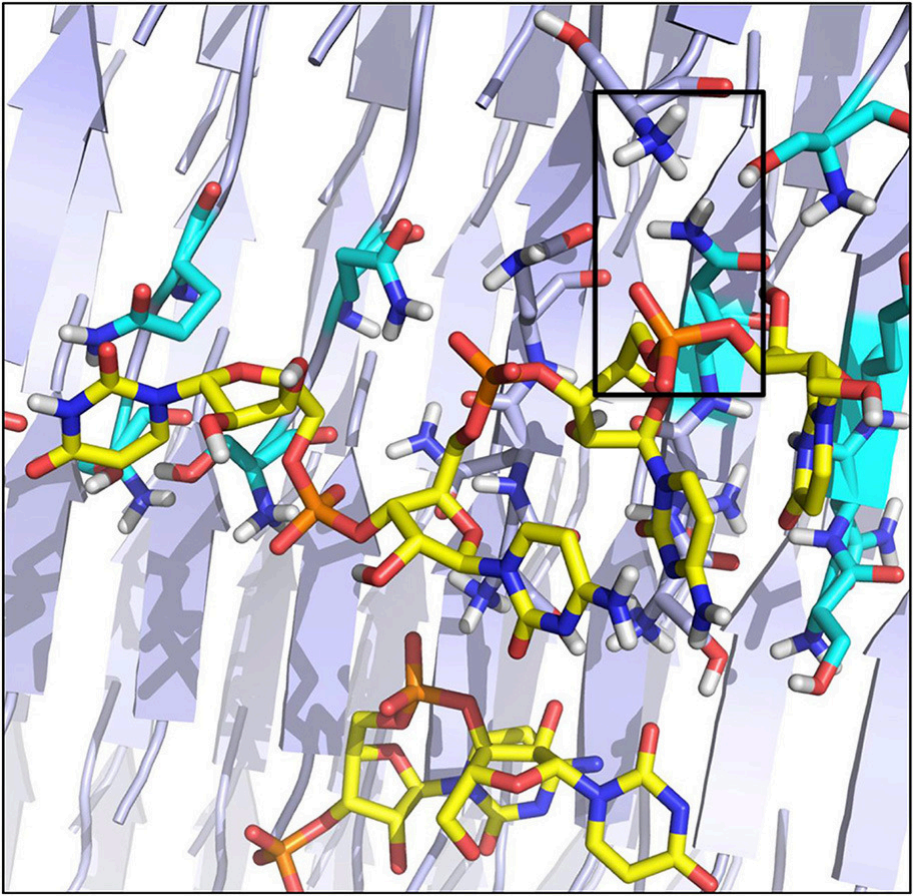
Organization of the RNA tetramer on the surface of the SNQNNF aggregate. Detail of the different h-bonds and electrostatic interactions on the surface. Electrostatic interactions are highlighted with a black box.

The higher affinity displayed by the SNQNNF-spine can be traced to the higher tendency of the positively charged N-terminals of the constitutive peptides to be exposed to the solvent. This, in turn, creates a large positively charged surface that is presented to the negatively charged backbone of the nucleotide tetramer (See Figures 3, 5). Complementary to this, the nucleobases can establish H-bonding interactions with the backbone groups displayed on the surface, favored also by the polarity of the exposed N side chains (Figure 5).

These structural, dynamical, and energetic analyses indicate that both amyloid models interact with the model RNA but with differential affinities, which are determined by the physicochemical properties of the peptide sequences. Our model is not refined enough to be able to tell whether the interactions of either sequence is permanent or transient, yet it provides information on the structural modulation that different sequences can impart on the RNA ligand: in this context, the role of charges, sidechains, H-bonds as well as the direction of RNA approach and docking appear to fine tune the details of molecular recognition.

### Formation of mixed RNA-amyloid oligomers

To investigate whether the presence of short RNA sequences may induce the formation of different amyloid oligomers, we extracted from the final frame of each RNA-amyloid crystal simulation the complex formed by the RNA stretch and all peptides within 12 Å from it. It is important to note that this type of model may be limited by the fact that we are considering a short stretch of RNA flanked by short peptidic sequences, extracted on the basis of the binding characterizations and of the distance criterion outlined above. Indeed, in an *in vitro* or *in vivo* setting, the situation may be significantly more complex, with different sequence peptide sequences binding around the RNA stretch or, complementarily, different RNA sequences binding amyloidogenic peptides. Notwithstanding these limitations, we suggest that the molecular information obtainable from this simplified model may represent a useful proxy to some of the main determinants of nucleic acid/amyloid interactions.

**Figure 6:**
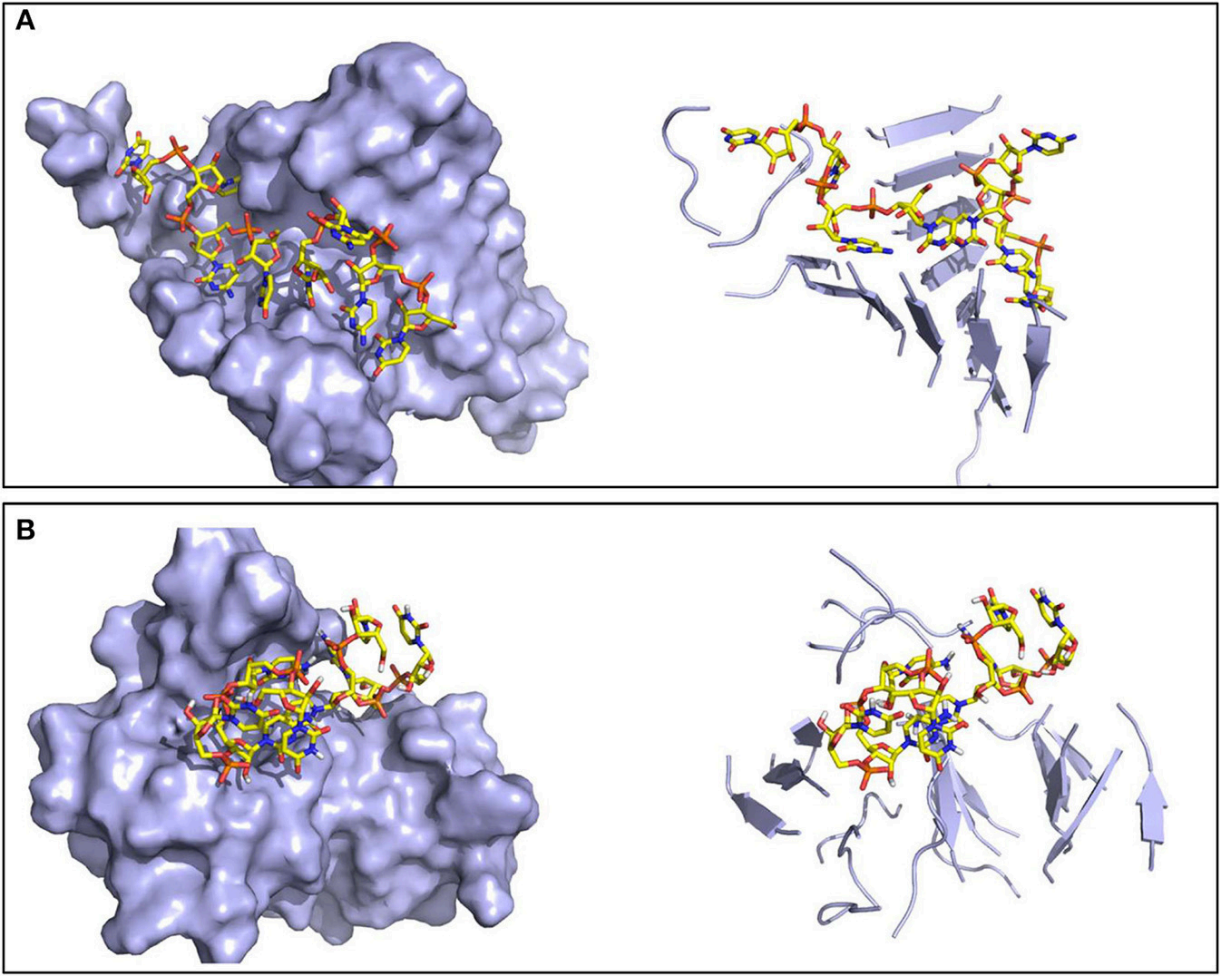
Representative structures of the oligomers formed by the RNA tetramer and peptide sequences. Surface (left) and secondary structure (right) representations of **(A)** the ordered oligomer formed with SNQNNF, and **(B)** the smaller and less organized oligomer formed with GYMLGS.

The resulting oligomers were subsequently simulated in explicit water, each for 200 ns. Structural differences between the two peptide sequences emerge. In the case of SNQNNF, its oligomers show in general high stability and the ability to form β-barrel structures with peptide stretches twisted along the principal axis and significant stacking of aromatic F side chains. These β-barrels or corkscrew-like assemblies have been found for amyloids and are considered as the toxic oligomeric entities given their capacity to form membrane pores (Serra-Batiste et al., 2016; Fusco et al., 2017; Sangwan et al., 2018) (Figure 6A). Moreover, a high degree of structural complementarity can be observed between the oligonucleotide and the peptidic aggregate (Figure 6A). The complexes built from the GYMLGS show different structure-dynamics profiles. The formation of a stable β-sheet/β-barrel type of oligomer is not noticeable and the peptides in the construct tend to be highly mobile and to give rise to structures that present to the RNA stretch a more limited contact surface (Figure 6B).

It is worth noting that while these data are based on a simplified model system, they may explain the differences in oligomer generation of distinct amyloid fibrils as a function of the sequence core. Fibrils such as those formed by SNQNNF are categorized as highly stable polar zippers based on the network of hyperpolarized intra-sheet hydrogen bonds formed between both side chain and main chain amides (Perutz et al., 1994; Mompeán et al., 2016). It is tempting to speculate that interaction with an RNA stretch may transiently disrupt such bonding and promote the formation of β-sheet rich oligomers/barrel like structures which may derail RNA-encoded messages to different cell compartments, ultimately causing cellular damage.

### Definition of possible pharmacophores for the discovery of new amyloid inhibitors

The obtained data can represent a step forward in identifying the molecular determinants of cores whose conformational and physicochemical properties are shared by potential lead-like amyloid inhibitors. Several studies have shown that various aromatic polycyclic compounds mimicking the described stacking interactions inhibit amyloid formation (Brumshtein et al., 2015). However, most of the molecular development following this rationale has failed to maintain their initial promises. We suggest that the decoration of aromatic cores with charged and hydrogen bonding groups with stereochemical relationships reminiscent of the ones observed in the simulations may provide pharmacophoric hypotheses for library screening. This pharmacophore design differs from the typical targeting of well-defined enzyme active sites. In this case, the ligand is described as a cloud of possible conformations that bind at different sites along the surface of a repetitive aggregate.

**Figure 7:**
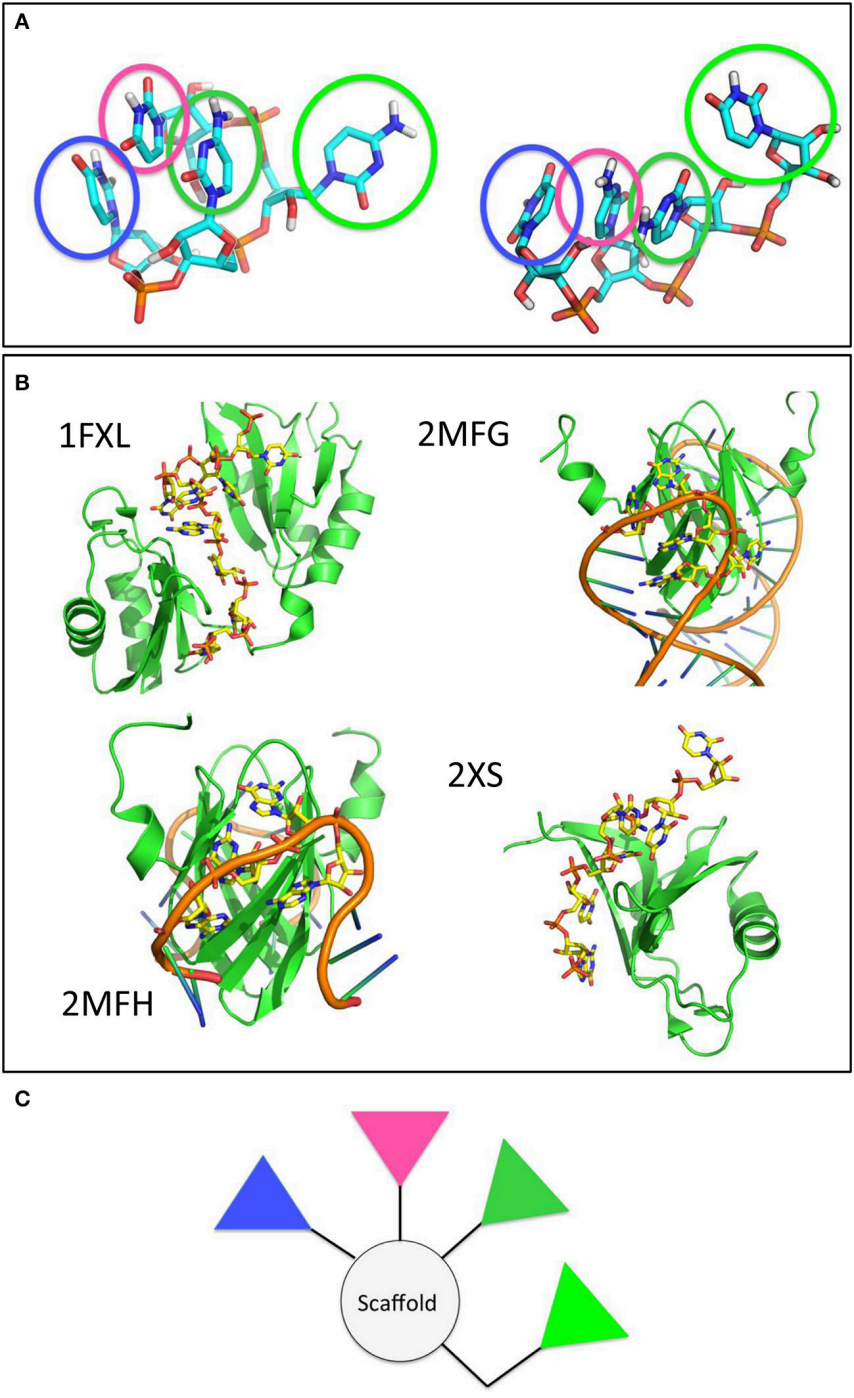
The main clusters populated by the model RNA stretch in contact with the amyloid surfaces are found in RNA-protein complexes and shape potential pharmacophores. **(A)** Main conformational clusters of the interacting RNA. The pyrimidinic groups responsible for contact and binding are highlighted by transparent circles. **(B)** Experimental structures of RNA-protein complexes. The structures of the proteins and RNA stretches are shown in cartoon. RNA side chains in contact with the protein surface, forming structures reminiscent of those observed in the previous panel are shown as yellow sticks. **(C)** A proposed model pharmacophore, whereby the colors of the different triangles correspond to the colors of the circles highlighted in **(A)**.

To proceed along these lines, we combined all the structures visited by RNA when in contact with either peptide sequence into a meta-trajectory. We then conducted a clustering analysis on each of the two meta-trajectories and extracted the central structure of the most populated cluster for each case (See Methods for details). The most representative conformations of RNA in complex with either peptide sequences are qualitatively similar, and are shown in Figure 7A.

To verify the potential validity of the observed RNA docking modes on the surfaces, we searched the Protein Data Bank for experimentally characterized structures of RNA-protein complexes. It is interesting to note that our simulation-based observation shown in Figure 7A are reminiscent of the structural features displayed by longer and more realistic RNA sequences when they form contacts with protein surfaces. Interestingly, the structures of the proteins reported are mostly β-sheet (Figure 7B).

In terms of drug design, we suggest that pharmacophore models may be designed by keeping into account the relative stereochemical relationships of the functional groups most responsible for binding the amyloid aggregates observed along the trajectories and by using different relative weights to keep into account the information on the persistence, stability and/or variability of the interactions established with the model aggregate. In this case, an ideal pharmacophore could be constituted by a central scaffold decorated with 3 to 4 aromatic polar moieties that mimic the pyrimidine groups (Figure 7C). Ideally, the latter should be linked to the scaffold by spacer groups that recapitulate their distances and orientations in space, or presented on nanoparticles to ensure a multivalent type of activity (Compostella et al., 2017).

## Conclusions

We have studied the recognition between a model RNA stretch and different amyloid fibrils by molecular dynamics simulations. To the best of our knowledge, this is one of the first reports of a possible interference or RNA-trapping mechanism exerted by an ordered amyloid fibril surface. We found that this interaction occurs and that for a common RNA stretch, the recognition is clearly peptide-sequence dependent. Interestingly, in the case of the SNQNNF fibril surface the interactions appear to be more stable and to favor the formation of β-barrel like complexes resembling the structures of toxic oligomers. On the basis of these results, specific amyloid sequences may sequester nucleic acid material on the surfaces their fibrillar and oligomeric assembly and derange the biochemical pathways in which RNA is used as an information carrying molecule. Moreover, we suggest that such initial sequence-structure-recognition relationships of the two different fibers may be a source of information for the design of compounds targeting the fibers or competing with RNA-amyloid attachment.

## Author contributions

MM designed research, ran simulations, wrote the paper. MG and GC designed research, wrote the paper.

### Conflict of interest statement

The authors declare that the research was conducted in the absence of any commercial or financial relationships that could be construed as a potential conflict of interest.
